# Supplementary material for: Structural basis for binding of RILPL1 to TMEM55B reveals a lysosomal platform for adaptor assembly through a conserved TBM motif
Source: bioRxiv. 2025 Aug 24:2025.08.19.670962. Preprint. [Version 1] doi: 10.1101/2025.08.19.670962 (PMC12393467; doi:10.1101/2025.08.19.670962)
Supplement: 1 [file NIHPP2025.08.19.670962v1-supplement-1.pdf]

bioRxiv preprint doi: https://doi.org/10.1101/2025.08.19.670962; this version posted August 24, 2025. The copyright holder for this preprint (which was not certified by peer review) is the author/funder, who has granted bioRxiv a license to display the preprint in perpetuity. It is made available under aCC-BY 4.0 International license.

Supplementary Table S1: Primary Antibodies

Antibody Target	Source	Catalogue Number (RRID)	Dilution
TMEM55B	Proteintech	23992-1-AP (AB_2879391)	1:1000
JIP4/ SPAG9	Cell Signaling Technology	5519 (AB_10828724)	1:1000
α-Tubulin	Cell Signaling Technology	3873 (AB_1904178)	1:10000
Halo	Promega	G9211 (AB_2688011)	1:1000
LRRK2 Total C-terminal	Neuromab	N241A/34 (AB_2877351)	1 µg/ml
Phospho-Rab8A (Pan-Thr-specific)	Abcam	ab230260 (AB_2814988)	1:1000
HA	Roche	3F10 (AB_2314622)	1:1000
GFP	ChromoTek	3H9 (AB_10773374)	1:1000
RILPL1	Abcam	Ab302492 (AB_2936945)	1:1000
TMEM55A	MRC PPU Reagents and Services, University of Dundee	DA241 (AB_2936451)	1 µg/ml

bioRxiv preprint doi: https://doi.org/10.1101/2025.08.19.670962; this version posted August 24, 2025. The copyright holder for this preprint (which was not certified by peer review) is the author/funder, who has granted bioRxiv a license to display the preprint in perpetuity. It is made available under aCC-BY 4.0 International license.

Supplementary Table S2: Secondary Antibodies

Antibody Target	Company	Catalogue number (RRID)	Dilution
IRDye 800CW Goat anti-Rabbit IgG	LI-COR	926-32211 (AB_621843)	1:25,000
IRDye 680CW Goat anti-Mouse IgG	LI-COR	926-68070 (AB_10956588)	1:25,000
IRDye 680CW Goat anti-Rat IgG	LI-COR	926-68074 (AB_10956736)	1:25,000
IRDye 800CW Donkey anti-Rabbit IgG	LI-COR	926-32213 (AB_621848)	1:25,000
IRDye 680LT Donkey anti-Mouse IgG	LI-COR	926–68 022 (AB_10715072)	1:25,000
IRDye 680LT Donkey anti-Goat IgG	LI-COR	926–68 024 (AB_10706168)	1:25,000

bioRxiv preprint doi: https://doi.org/10.1101/2025.08.19.670962; this version posted August 24, 2025. The copyright holder for this preprint (which was not certified by peer review) is the author/funder, who has granted bioRxiv a license to display the preprint in perpetuity. It is made available under aCC-BY 4.0 International license.

Supplementary Figure S1

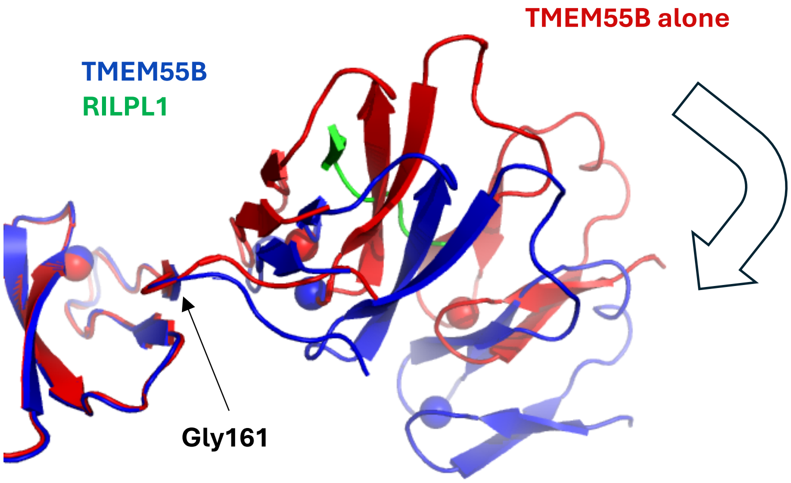

A. Ribbon models of TMEM55B alone and in complex with RILPL1. The asymmetric unit is a dimer in both crystals. Molecule B has been superposed (left), and molecule A shows a relative rotation of approximately 30°. The hinge for this difference is Gly161, whose ϕ angle rotates by 37°. The list of ϕ/Ψ are below:
TMEM55B alone = L160 (-87.4°, 9.8°), G161 (-176.1°, 158.9°), P162 (-67.5°, 157.5°);
TMEM55B complex = L160 (-106.5 °, 26.3°), G161 (-139°, 164.8°), P162 (-80.7 °, 164.7

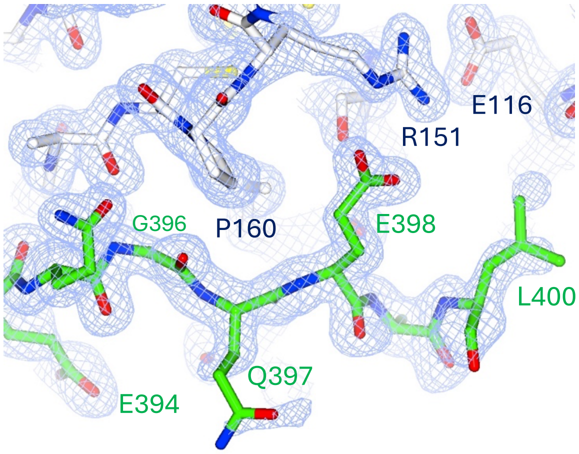

B. A section of the electron density around the TBM motif from the crystal structure of TMEM55B/RILPL1 complex. The RILPL1 peptide is green, and the TMEM55B is grey. The 2Fo-Fc map is contoured at 1σ density.

bioRxiv preprint doi: https://doi.org/10.1101/2025.08.19.670962; this version posted August 24, 2025. The copyright holder for this preprint (which was not certified by peer review) is the author/funder, who has granted bioRxiv a license to display the preprint in perpetuity. It is made available under aCC-BY 4.0 International license.

Supplementary Figure S2

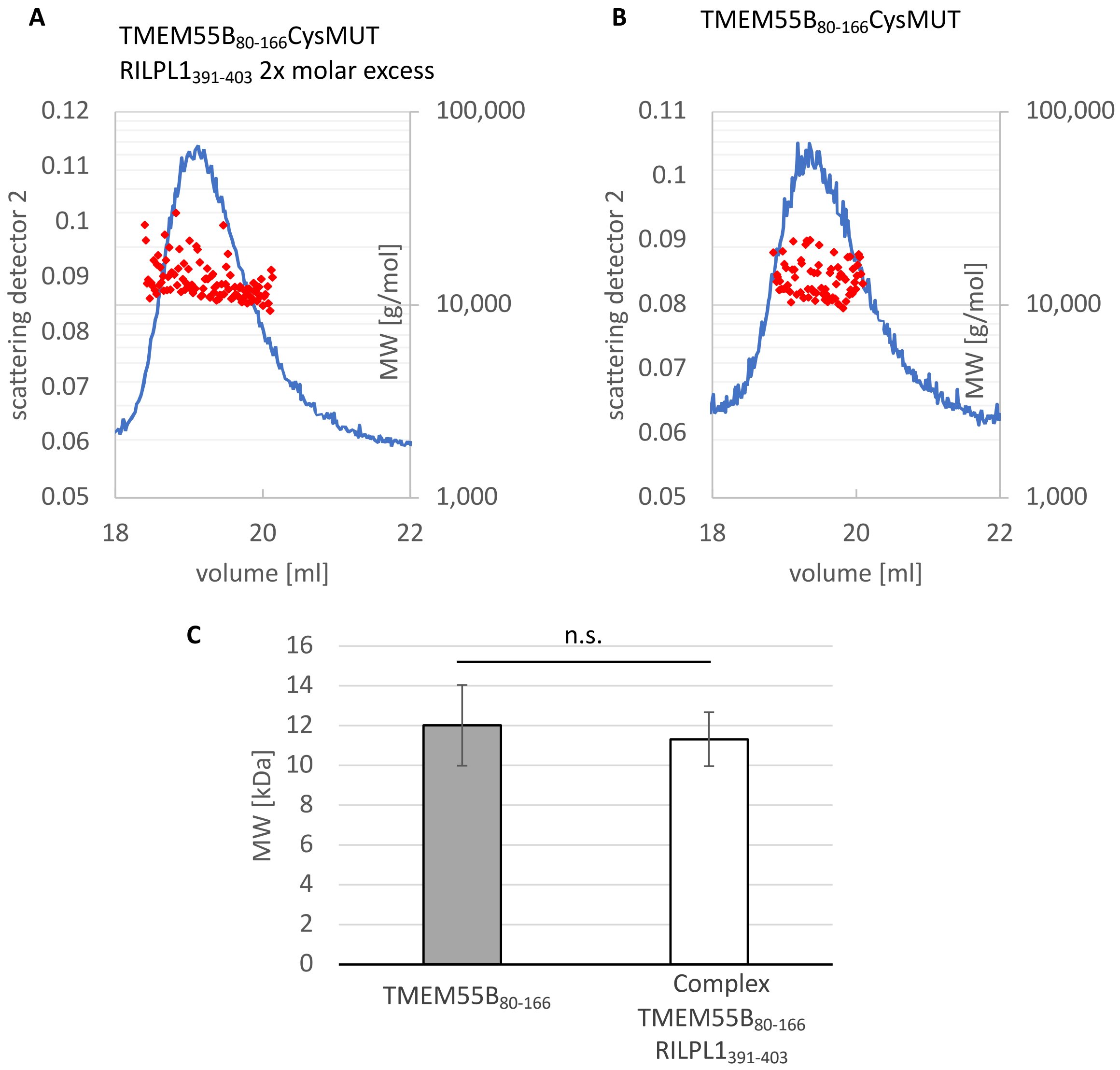

Figure S2: Size exclusion chromatography coupled to light scattering was performed to compare TMEM55B$_{80\text{-}166}$2CysMUT alone with a sample pre-incubated with peptide RILPL1$_{391\text{-}403}$. Samples were injected onto a Superdex75 10/300 column coupled to the Wyatt systems described in Methods. Absolute mass measurements were calculated at 1 second intervals. **(A)** and **(B)** show representative runs of TMEM55B/RILPL1 and TMEM55B alone, respectively. **(C)** Bar plot of the calculated molecular weights from 3 independent experiments. For each independent experiment, the value of the molecular weight was calculated as the average over the peaks (red dots in **A** and **B**).

bioRxiv preprint doi: https://doi.org/10.1101/2025.08.19.670962; this version posted August 24, 2025. The copyright holder for this preprint (which was not certified by peer review) is the author/funder, who has granted bioRxiv a license to display the preprint in perpetuity. It is made available under aCC-BY 4.0 International license.

Suppemental Figure S3

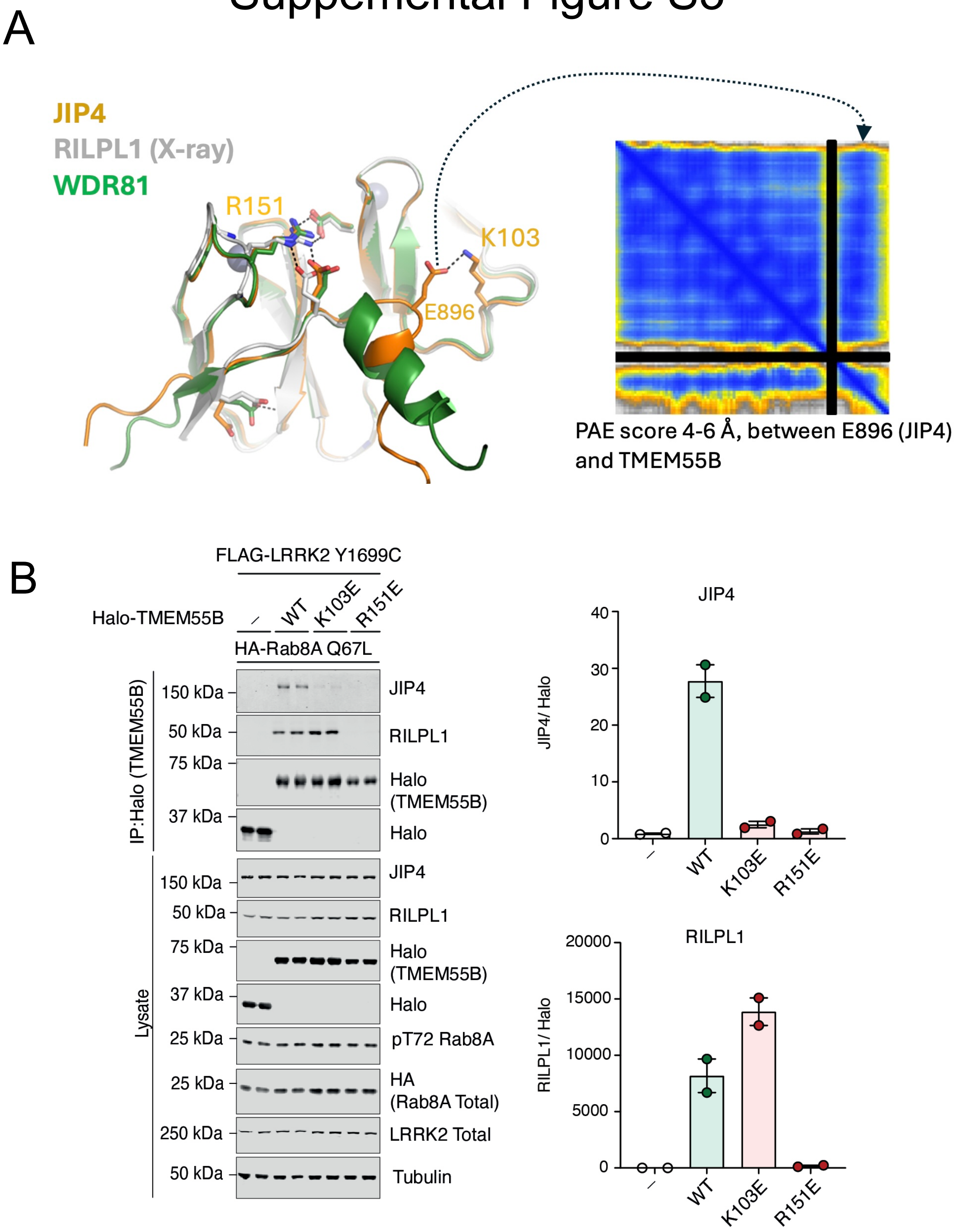

Figure S3: (A) AlphaFold3 predictions of TMEM55B in complex with JIP4 and WDR81. Peptide predictions are aligned onto the X-ray structure of RILPL1 peptide. The panel on the right shows a graphic of PAE scores from the JIP4/TMEM55B model, visualized using ChimeraX (ref). JIP3 was not included in the alignment because AlphaFold3 failed to yield a reasonable model. (B) HEK293 TMEM55B/A double KO cells were transiently transfected with HA-Rab8(Q67L) and FLAG-LRRK2(Y1699C, kinase-active mutant), in the presence of WT, K130E and R151E variants of Halo-TMEM55B. Halo-IP was performed and analyzed by quantitative immunoblot analysis using the LiCOR Odyssey CLx Western blot imaging system and indicated antibodies. Quantitation of immunoblotting data are dhown as mean +/- SEM (ImageStudio Lite version 5.2.5).

bioRxiv preprint doi: https://doi.org/10.1101/2025.08.19.670962; this version posted August 24, 2025. The copyright holder for this preprint (which was not certified by peer review) is the author/funder, who has granted bioRxiv a license to display the preprint in perpetuity. It is made available under aCC-BY 4.0 International license.

Supplementary Figure S4

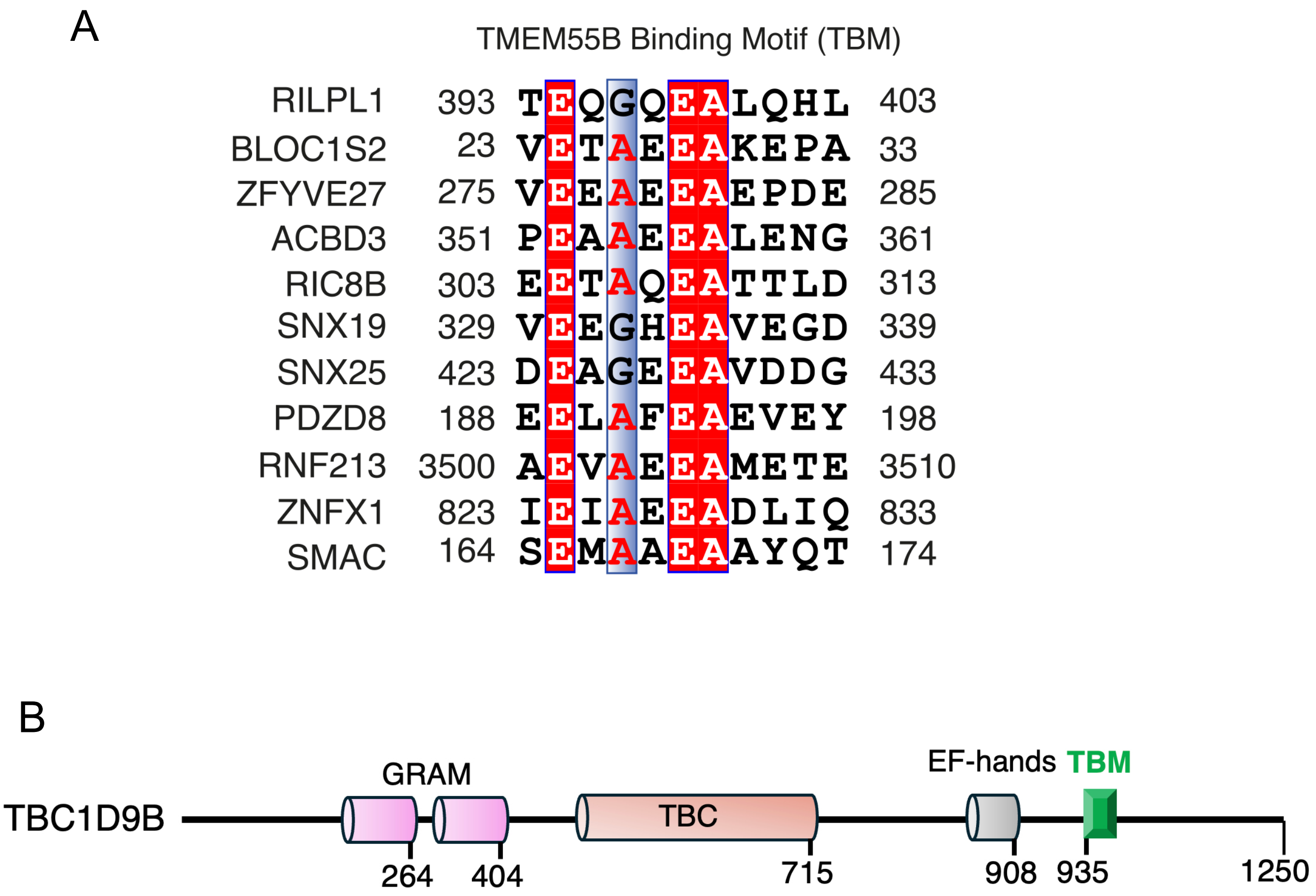

Figure S4: (A) Potential TBMs from interactors identified by mass spectrometry. These proteins have not been verified by independent experiments. (B) Domain organization of TBC1D9B with location of the TBM indicated in green.

bioRxiv preprint doi: https://doi.org/10.1101/2025.08.19.670962; this version posted August 24, 2025. The copyright holder for this preprint (which was not certified by peer review) is the author/funder, who has granted bioRxiv a license to display the preprint in perpetuity. It is made available under aCC-BY 4.0 International license.

Supplementary Figure S5

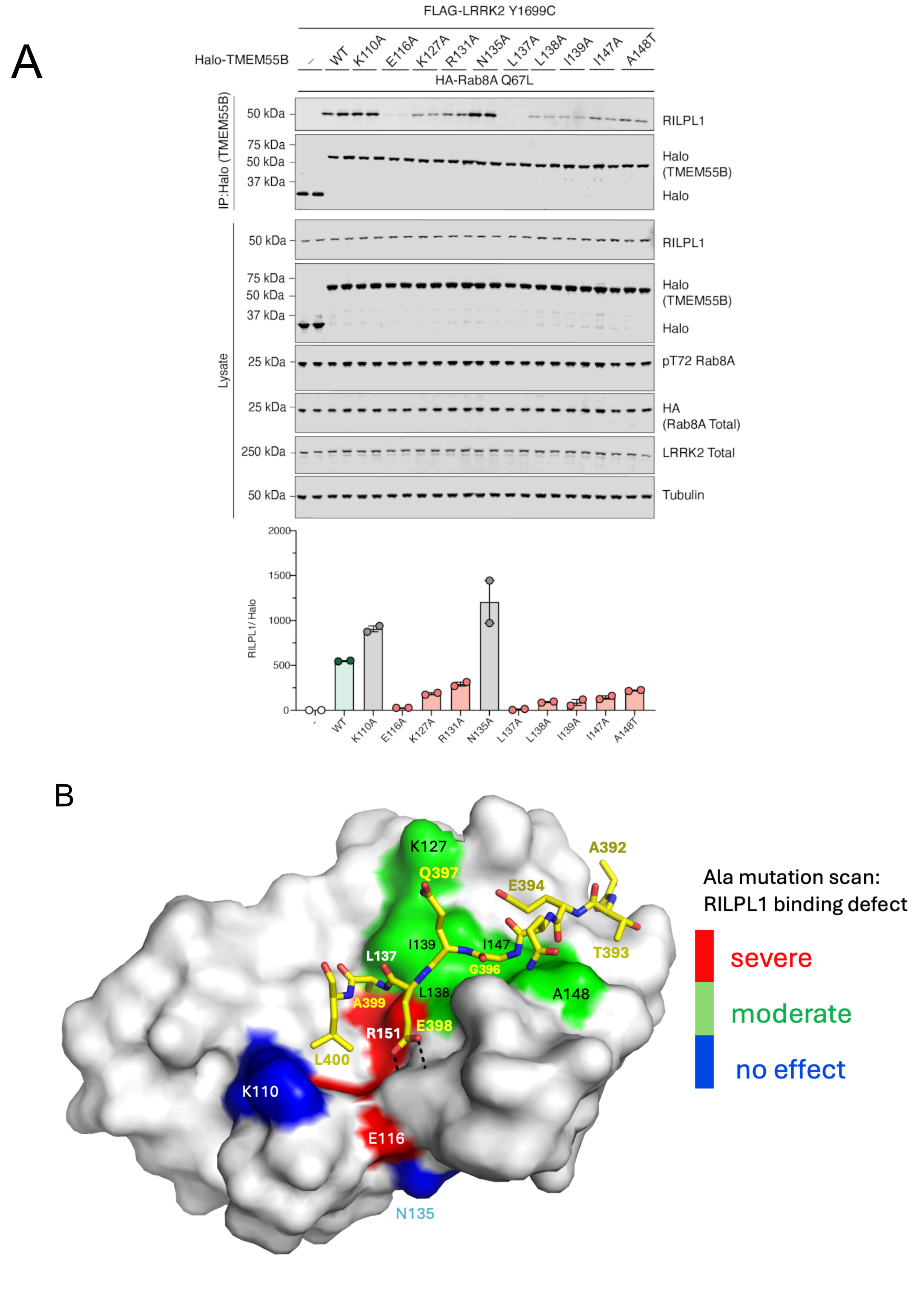

Figure S5: **(A)** HEK293 TMEM55B/A double knockout cells were transiently transfected with HA-Rab8a(Q67L GTP-bound mutant) and FLAG-LRRK2(Y1699C kinase-active mutant) either in the presence of Halo-TMEM55B WT or ten different mutants (K110A, E116A, K127A, R131A, N135A, L137A, L138A, I139A, I147A, A148T) at the TMEM55B interface. Halo immunoprecipitation was performed and analyzed by quantitative immunoblot analysis using the LI-COR Odyssey CLx Western blot imaging system and indicated antibodies. Quantitation of immunoblotting data (performed using ImageStudioLite software version 5.2.5, RRID:SCR_013715) is shown as mean ±SEM. **(B)** Surface map of TMEM55B in complex with stick model of RILPL1 peptide (yellow). Residues on TMEM55B surface that were mutated to alanine are colored red (negatively charged), blue (positive), cyan (polar) and green for non-polar side chains. The key electrostatic interaction interaction between E398^{RL1} and R151^{TM} is emphasized with dashed black line reaching out to the grey surface contours of arginine.
